# Register of Dentists

**Published:** 1897-10

**Authors:** 


Register of Dentists. By R. L. Polk & Co. Published in Detroit, Baltimore and Chicago. Second edition.

This is the most complete register of dentists that has ever been prepared and made public. It gives the names, location and status of the dentists in every city, town and hamlet in the United States; it indicates those who are graduates, those who are licensees under a State law, as well as those who are practicing without either of those endorsements. The college is indicated from which a graduate received his diploma; the number of dentists in each State; the number and name of dental societies of each State and the dental law of each State. There is also a complete alphabetical list of the dentists of the country, and in addition it contains a classified list of the persons and firms engaged in the manufacture and sale of dental goods.

This is an invaluable work to all dentists who assume to be anything but mere plodders in their profession; and ought to be in the hands of every one who has any ambition to know the character of his profession, and especially those who have frequent occasion for communication and contact with his professional brethren.

The work can be obtained of the publishers or through any dental depot.

